# Correction: Felsenstein M. et al. Perineural Invasion in Pancreatic Ductal Adenocarcinoma (PDAC): A Saboteur of Curative Intended Therapies? *J. Clin. Med.* 2022, *11*, 2367

**DOI:** 10.3390/jcm12216947

**Published:** 2023-11-06

**Authors:** Matthäus Felsenstein, Flora Lindhammer, Mathilde Feist, Karl Herbert Hillebrandt, Lea Timmermann, Christian Benzing, Brigitta Globke, Dario Zocholl, Mengwen Hu, Uli Fehrenbach, Bruno Valentin Sinn, Uwe Pelzer, Igor Maximillian Sauer, Johann Pratschke, Thomas Malinka

**Affiliations:** 1 Department of Surgery, Campus Charité Mitte/Campus Virchow-Klinikum, Charité-Universitätsmedizin Berlin, Augustenburger Platz 1, 13353 Berlin, Germany; 2 Berlin Institute of Health (BIH), Charité-Universitätsmedizin Berlin, Anna-Louisa-Karsch-Str. 2, 10178 Berlin, Germany; 3 Institute of Biometry and Clinical Epidemiology, Campus Charité Mitte, Charité-Universitätsmedizin Berlin, 10117 Berlin, Germany; 4 Department of Radiology, Campus Charité Mitte/Campus Virchow-Klinikum, Charité-Universitätsmedizin Berlin, 10117 Berlin, Germany; 5 Institute of Pathology, Campus Charité Mitte, Charité-Universitätsmedizin Berlin, 10117 Berlin, Germany; 6 Medical Department, Division of Hematology, Oncology and Tumor Immunology, Campus Charité Mitte/Campus Virchow-Klinikum, Charité-Universitätsmedizin Berlin, 10117 Berlin, Germany

## 1. Error in Table

In the original publication [1], there was an error in Tables 1 and 2, as published. There was an inaccuracy in the data transfer from the primary Excel files. The corrected tables can be found below.

## 2. Text Correction

The authors state that the scientific conclusions are unaffected. Based on the modified Table 1 and Table 2, the following sentences need to be adjusted:

1. Section 3, Section 3.1, Paragraph 1, from fifth to seventh sentences should be: “There were 22 female (55%) and 18 male (45%) patients negative for perineural invasion (Pn0 group) with a median age of 64.7 years (range 35–84). The group of patients positive for perineural invasion (Pn1 group) consisted of 242 female (45.6%) and 289 male (54.4%) individuals with a median age of 65.6 years (range 37–83). Tumor location in over 70% of both groups was the head of the pancreas (Pn0 group: 70 %, Pn1 group: 75.1%), followed by the pancreatic tail (Pn0 group: 17.5%, Pn1 group: 11.9%) and pancreatic body (Pn0 group: 7.5%, Pn1 group: 8.1%).”
2. Section 3, Section 3.2, Paragraph 2, the last two sentence should be: “There was a significant difference between the BMI of the Pn0 and Pn1 group (*p* = 0.05). Pre-operative tumor markers (CA19-9, CEA) that well reflect the overall tumor burden, including micro-metastases, did not reveal strong correlations.”
3. Section 3, Section 3.3, the first sentence should be: “Studying long-term survivors (LTS with survival >5 years) in our study cohorts, we discriminated a significantly increased number of LTS in the Pn0 group compared to Pn1 patients (*p* = 0.04).”
4. Section 3, Section 3.2, Paragraph 1, from third to fifth sentences should be: “In our cohort, 30% of Pn0 patients presented with early stage pT1 tumors (Pn0, pT1: 30%; Pn0, pT2: 50%; Pn0, pT3: 20%; Pn0, pT4: 0%), while only 10.5% of Pn1 patients presented with pT1 tumors (Pn1, pT1: 10.5%; Pn1, pT2: 57.1%; Pn1, pT3: 27.9%; Pn1, pT4: 4.5%; *p* = 0.007). The majority of Pn1 tumors showed lymph node metastasis (Pn1, pN+: 75.5% versus Pn1, pN0: 24.5%), while lymph node infiltration occurred only in 40% of Pn0 tumors (Pn0, pN+: 40% versus Pn0, pN0: 60%; *p* < 0.001). This also significantly correlated with lymphatic invasion; 44.1% of the Pn1 tumors were positive for lymphatic invasion (Pn1 L1: 44.1%; Pn1 L0: 55.9%).”
5. Section 3, Section 3.2, Paragraph 1, the last two sentence should be: ”There also appeared to be a detectable difference in the grading of Pn1 tumors compared to Pn0 tumors with a shift toward less-differentiated tumors in the Pn1 group (Pn1 G1: 3%; Pn1 G2: 60.5%; Pn1 G3: 36.5%—Pn0 G1: 10%; Pn0 G2: 67.5%; Pn0 G3: 22.5%; *p* = 0.03). However, vascular invasion and resection margin did not significantly correlate with either Pn0 or Pn1 tumors.”

For a more complete understanding and clarification of our conducted analyses and exclusion criteria, we needed to modify the following sentence (Section 2, Section 2.1, the last two sentences):

“Patients with incomplete medical history documentation, R-status, PNI status and tumor stage were excluded. Patients with in-hospital mortality (<30 days survival) or who were lost to follow-up during that time were excluded for analyses of time to event outcomes (Kaplan–Meier curves and Multivariate Cox regression on overall- and disease-free survival).”

In addition, we detected an error as we included patients starting from March 2008, not January. We request to modify the sentence as follows (Section 2, Section 2.1, the first sentence):

“Patients undergoing curative intended surgical resection for PDAC at the Department of Surgery, Campus Charité Mitte|Campus Virchow, Charité—Universitätsmedizin, Berlin, Germany, between March 2008 and December 2019 were included.”

For a validation of our results, we have used additional statistical software, now also included in the Section 2, Section 2.3, the first sentence:

“For statistical analysis, the statistical software R (The R Foundation, Version 4.0.0), SPSS (IBM SPSS Statistic, Version 28.0) and Prism (Graph Pad Software, La Jolla, CA, USA) were used.”

Section 4, Paragraph 5, the third sentence should be: “During the entire study period, 11.2% of the patients received neoadjuvant treatment.”

A correction has been made to Funding section:

**Funding:** Matthäus Felsenstein, Karl-Herbert Hillebrandt and Brigitta Globke are participants in the BIH-Charité Clinician Scientist Program, funded by the Charité–Universitätsmedizin Berlin and the Berlin Institute of Health.

We also modified the Supplementary Table S1 for a better overview of adjuvant chemotherapy regimen across all patients, which was missing in the previously published version. There were no modifications needed in the table content/numbers.

The authors state that the scientific conclusions are unaffected. This correction was approved by the Academic Editor. The original publication has also been updated.

## Figures and Tables

**Table 1:** Patient characteristics of entire study cohort, Pn0 and Pn1 groups.

	Total	%	Pn0	%	Pn1	%	Statistics *
**Cases**	571		40		531		
**Age** (years)	65.6 (+/−11.1)		64.7 (+/−10.5)		65.6 (+/−10.8)		*p* = 0.58
**Sex**	female 264	46.2	female 22	55	female 242	45.6	*p* = 0.26
	male 307	53.8	male 18	45	male 289	54.4	
**Body mass index** (BMI) (in kg/m^2^)	25.1 (+/−4.3)		23.6 (+/−4.0)		25.2 (+/−4.3)		***p* = 0.05**
**Diabetes mellitus** (DM)							
DM I	19	3.3	0	0	19	3.6	*p* = 0.39
DM II	116	20.3	7	17.5	109	20.5	*p* = 0.84
**Beta blocker**							*p* = 0.22
ß1 selective	168	29.4	9	22.5	159	29.9	
Non-selective	12	2.1	0	0	12	2.3	
**Carbohydrate-antigen 19-9** (in U/mL)	846.3 (+/−3014)		453.6 (+/−1303)		888.5 (+/−3142)		*p* = 0.49
**Carcinoembryonic antigen** (in µg/L)	18.8 (+/−67.9)		4.5 (+/−3.2)		20.0 (+/−72.9)		*p* = 0.28
**Tumor entity**							*p* = 0.77
Head	427	74.8	28	70	399	75.1	
Tail	70	12.3	7	17.5	63	11.9	
Body	46	8.1	3	7.5	43	8.1	
Uncinate	28	4.9	2	5	26	4.9	
**Surgical procedure**							***p* = 0.05**
PPPD **	363	63.6	20	50	343	64.6	
Whipple	29	5.1	5	12.5	24	4.5	
Total	94	16.5	6	15	88	16.5	
Distal	85	14.9	9	22.5	76	14.3	
**Chemotherapy**							
Pre-operative	64	11.2	8	20	56	10.5	*p* = 0.11
Post-operative	353	61.8	28	70	325	61.2	*p* = 0.31
**Clinical Outcome**							
Follow-up (months)	17.2		24.2		16.6		***p* = 0.04**
30-day mortality	28	5.2	0	0	28	5.6	*p* = 0.25
Death	383	78.8	15	41.7	368	81.8	***p* < 0.001**
Alive	103	18.0	21	52.5	82	15.4	***p* < 0.001**
Lost to follow-up	85	14.9	4	10	81	15.3	*p* = 0.49
LTS *** (>5 years)	19	3.3	4	10	15	2.8	***p* = 0.04**
Recurrence							
Yes	167	29.2	11	27.5	156	29.4	*p* = 0.86
No	404	70.8	29	72.5	375	70.6	

* Fisher’s exact test for categorical variables and Student’s *t*-test for continuous variables. ** Pylorus preserving pancreaticoduodenectomy. *** Long-term survivors (>5 years).

**Table 2:** Histopathological assessment of tumors in Pn0 and Pn1 groups.

	Total	%	Pn0	%	Pn1	%	Statistics *
**Cases**	571		40		531		
**Tumor stage**							***p* = 0.007**
pT1 (<2 cm)	68	11.9	12	30	56	10.5	
pT2 (2–4 cm)	323	56.6	20	50	299	57.1	
pT3 (>4 cm)	176	27.3	8	20	148	27.9	
pT4 (vessel infiltration)	24	4.2	0	0	24	4.5	
**Lymph node metastasis**							***p* < 0.001**
N−	154	27	24	60	130	24.5	
N+	417	73	16	40	401	75.5	
**Distant metastasis**							*p* = 0.3
M0	510	89.3	38	95	472	88.9	
M1	61	10.7	2	5	59	11.1	
**Histologic grade**							***p* = 0.03**
G1	20	3.5	4	10	16	3	
G2	348	60.9	27	67.5	321	60.5	
G3	203	35.6	9	22.5	194	36.5	
**Resection margin**							*p* = 0.06
R0	359	62.9	31	77.5	328	61.8	
R1	212	37.1	9	22.5	203	38.2	
**Vascular invasion**							*p* = 0.09
V0	467	81.8	37	92.5	430	81	
V1	104	18.2	3	7.5	101	19	
**Lymphatic invasion**							***p* < 0.001**
L0	330	57.8	33	82.5	297	55.9	
L1	241	42.2	7	17.5	234	44.1	

* Fisher’s exact test for categorical variables and Student’s *t*-test for continuous variables.
