# Supplementary material for: Perineural Invasion in Pancreatic Ductal Adenocarcinoma (PDAC): A Saboteur of Curative Intended Therapies?
Source: J Clin Med. 2022 Apr 23;11(9):2367. doi: 10.3390/jcm11092367 (PMC9103867; doi:10.3390/jcm11092367)
Supplement: Supplementary file 1 [file jcm-11-02367-s001.zip › jcm-1658925-supplementary.pdf]

Supplementary Materials:

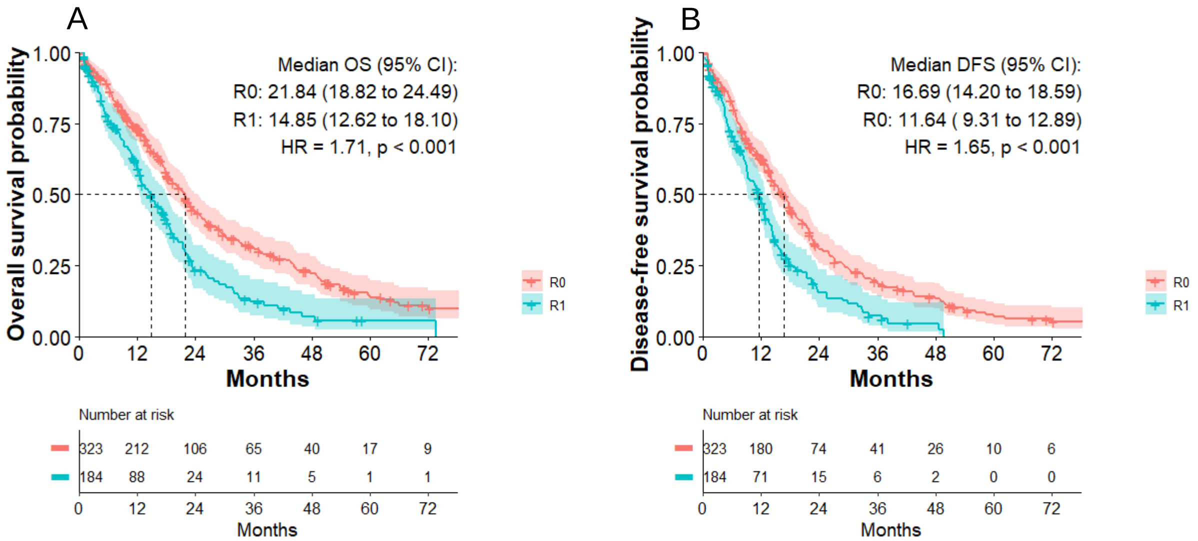

Figure S1: Overall- and disease-free survival of patients with R0 or R1 margin status. **A:** Overall survival of R0 and R1 margin status. **B:** Disease-free survival of R0 and R1 margin status.

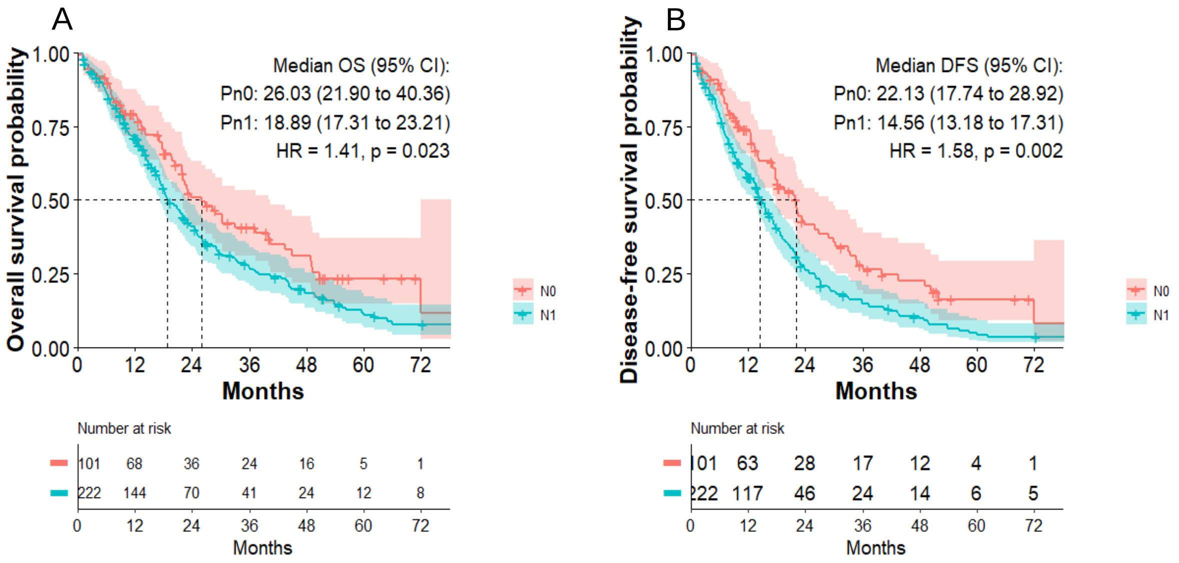

Figure S2: Overall- and disease-free survival of margin negative patients with either N0 or N1 status. **A:** Overall survival of R0 N0 and R0 N1 group. **B:** Disease-free survival of R0N0 and R0N1 group.

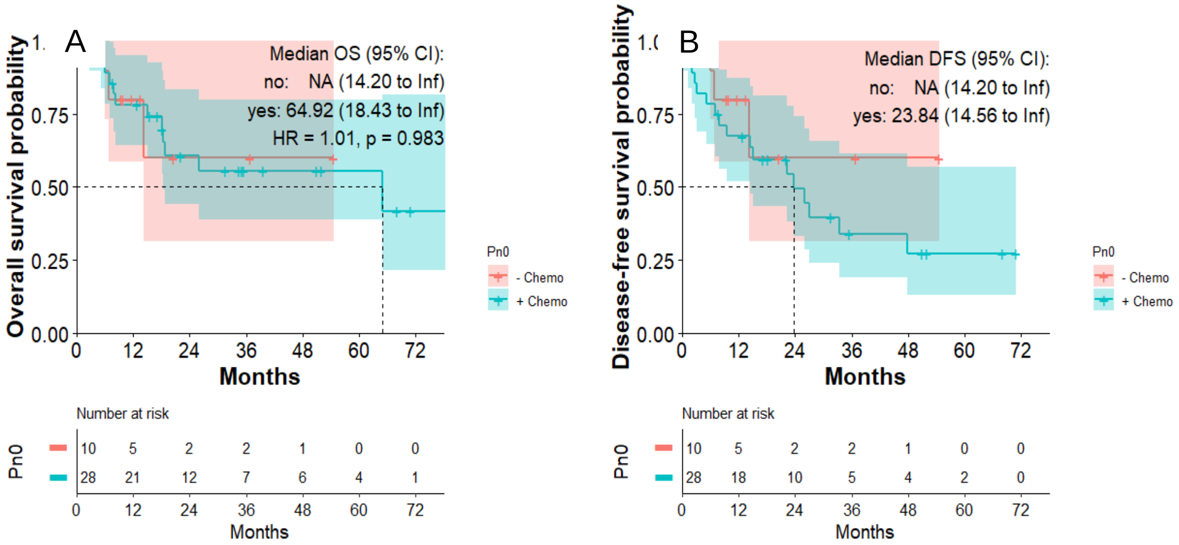

Figure S3: Overall survival and disease-free survival of patients with R0 Pn0 tumors with or without postoperative chemotherapy. **A:** Overall survival of patients with Pn0 tumors with or without postoperative chemotherapy. **B:** Disease-free survival of patients with Pn0 tumors with or without postoperative chemotherapy.

Table S1. Institutionell Chemotherapy regimen

Chemotherapy	Total 571	%	Pn0 40	%	Pn1 531	%
**Neoadjuvant**						
No	507	89	32	80	475	89.4
Yes	64	11	8	20	56	10.5
Platin-based	36		5		31	
Gemcitabin-based	39		3		36	
Other	2		0		2	
**Adjuvant**						
No			12	30	206	38.7
Yes			28	70	325	61.2
Platin-based			12		91	
Gemcitabin-based			21		263	
Other			1		9	
